# Blood Group Antigen Expression in Blood and Tumor in Relation to Survival Outcomes in Resected Pancreatic Cancer, Overall and by Adjuvant Chemotherapy Regimens

**DOI:** 10.1245/s10434-025-17289-7

**Published:** 2025-05-02

**Authors:** Yosuke Inoue, Manabu Takamatsu, Yohei Masugi, Tatsunori Suzuki, Tsuyoshi Hamada, Satoru Abe, Kensuke Hara, Yoshikuni Kawaguchi, Kosuke Kobayashi, Aya Maekawa, Yousuke Nakai, Naoki Sasahira, Tsuyoshi Takeda, Mariko Tanaka, Yosuke Uematsu, Sho Uemura, Tetsuo Ushiku, Mitsuhiro Fujishiro, Kengo Takeuchi, Minoru Kitago, Kiyoshi Hasegawa, Yu Takahashi, for the GTK Pancreatic Cancer Study Group in Japan, Satoko Baba, Shuhei Ishii, Motoyoshi Iwakoshi, Kikuko Kaji, Kei Sakuma, Noriko Koga

**Affiliations:** 1 Department of Hepatobiliary and Pancreatic Surgery, Cancer Institute Hospital, Japanese Foundation for Cancer Research, Tokyo, Japan; 2 Division of Pathology, The Cancer Institute of Japanese Foundation for Cancer Research, Tokyo, Japan; 3 Department of Pathology, Cancer Institute Hospital, Japanese Foundation for Cancer Research, Tokyo, Japan; 4 Department of Pathology, Keio University School of Medicine, Tokyo, Japan; 5 Division of Diagnostic Pathology, Keio University School of Medicine, Tokyo, Japan; 6 Department of Gastroenterology, Graduate School of Medicine, The University of Tokyo, Tokyo, Japan; 7 Department of Hepato-Biliary-Pancreatic Medicine, Cancer Institute Hospital, Japanese Foundation for Cancer Research, Tokyo, Japan; 8 Hepato-Biliary-Pancreatic Surgery Division, Department of Surgery, Graduate School of Medicine, The University of Tokyo, Tokyo, Japan; 9 Department of Endoscopy and Endoscopic Surgery, The University of Tokyo Hospital, Tokyo, Japan; 10 Department of Pathology, Graduate School of Medicine, The University of Tokyo, Tokyo, Japan; 11 Department of Surgery, Keio University School of Medicine, Tokyo, Japan

**Keywords:** Blood group antigens, Cohort studies, Mortality, Pancreatectomy, Pancreatic ductal adenocarcinoma

## Abstract

**Background:**

Few comprehensive studies have examined the associations of the ABO blood group with survival outcomes for patients with resected pancreatic cancer, overall and by adjuvant chemotherapy regimens.

**Methods:**

This multicenter study enrolled 1153 patients with resected pancreatic cancer. The hazard ratios (HRs) for disease-free and pancreatic cancer-specific survival were calculated with adjustment for potential confounders, including *KRAS* mutation and CDKN2A (p16), TP53, and SMAD4 expression, using the Cox proportional hazards regression model. Blood group antigen expression in tumors was immunohistochemically assessed.

**Results:**

The ABO blood group was not associated with disease-free or pancreatic cancer-specific survival (*P* > 0.90). For pancreatic cancer-specific survival, blood groups A, B, and AB had multivariable HRs of 0.97 (95% confidence interval [CI], 0.81–1.15), 1.03 (95% CI, 0.83–1.26), and 0.99 (95% CI, 0.76–1.30), respectively (vs. O). The associations between ABO blood group and disease-free and pancreatic cancer-specific survival differed according to the adjuvant chemotherapy regimens (*P*_interaction_ = 0.011 and 0.008, respectively). For the patients without chemotherapy, the HRs for disease-free survival were 0.99 (95% CI, 0.69–1.41) for blood group A, 1.65 (95% CI, 1.09–2.48) for blood group B, and 1.79 (95% CI, 1.01–3.17) for blood group AB, (vs. O). For the patients receiving S-1-based chemotherapy, blood group AB (vs. O) exhibited a reverse association (HR, 0.63; 95% CI, 0.39–1.00). Similar interactions were observed when blood group antigen expression in tumors was analyzed.

**Conclusions:**

The ABO blood group is not a prognostic biomarker in resected pancreatic cancer overall but may predict the effectiveness of adjuvant chemotherapy.

**Supplementary Information:**

The online version contains supplementary material available at 10.1245/s10434-025-17289-7.

Pancreatic cancer (PC) currently is the third leading cause of cancer-related deaths in the United States, with a 5-year survival rate of approximately 10%.^1^ Recent advances in multimodal anti-cancer treatment with new chemotherapy regimens and surgical methods have prolonged long-term survival after resection.^2–4^ However, survival prolongation has been insufficient, partly due to a lack of reliable biomarkers for chemotherapy response and overall prognosis.

In clinical practice, carbohydrate antigen 19-9 (CA19-9) is a universally available biomarker that aids in decision-making regarding chemotherapy and surgery as well as in predicting and evaluating tumor response to chemotherapy.^5–8^ However, CA19-9 has inherent limitations, characterized by false-negatives for Lewis antigen-negative individuals with no CA19-9 expression (approximately 10% of the population)^9^ and false-positives for patients with obstructive jaundice.^10^ Therefore, additional biomarkers are needed to risk-stratify patients with resected PC and thereby optimize treatment strategies.

The ABO blood group has been implicated in the risk for pancreatic carcinogenesis, with supporting evidence from cohort and genome-wide association studies.^11–15^ The ABO blood group classification categorizes blood groups based on the presence or absence of A and B antigens on red blood cell surfaces, which also are expressed on the surface of several other cells, potentially provoking cancer development in the corresponding organs.^16–18^ Recent reports have investigated the relationship between the ABO blood group and cancer prognosis, with several studies suggesting a prognostic role for the blood group in PC.^19–21^ A surgical series of patients with PC indicated that longer survival was associated with blood group O for patients receiving specific adjuvant chemotherapy regimens,^22^ but such a differential treatment response with respect to the ABO blood group has not been validated in large populations. If a specific association with PC prognosis is confirmed, the ABO blood group may serve as an additional biomarker for treatment decision-making and surveillance protocols.

We hypothesized that the ABO blood group might be associated with survival for patients with resected PC, which might be augmented for patients receiving specific adjuvant chemotherapy regimens. Using a large multicenter database of resected PC with comprehensive data on clinical, pathologic, and molecular characteristics, we examined the ABO blood group and its interaction with adjuvant chemotherapy in relation to survival outcomes. Previous reports noted a substantial frequency of aberrant expression of A and B antigens in tumor cells, potentially altering tumor biology or metastatic potential in various cancers, including PC.^18,23–28^ Therefore, we additionally examined blood group antigen expression patterns in tumor tissues using artificial intelligence-based image analysis for immunohistochemistry (IHC) in a subset of patients and performed similar survival analyses focusing on the tumor expression levels of these antigens.

## Methods

### Study Population and Data Collection

**Fig. 1:**
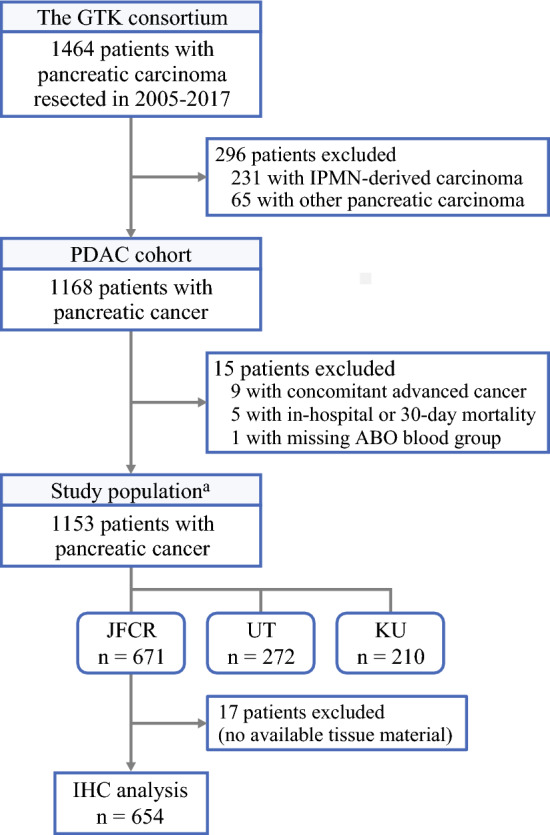
Flow diagram showing selection of patients with resected pancreatic cancer in a multi-institutional cohort. ^a^For analyses of disease-free survival, the study further excluded 60 patients with a resected metastatic lesion, R2 resection margin, or no available cross-sectional imaging after the index surgery. IHC, immunohistochemistry; IPMN, intraductal papillary mucinous neoplasm; JFCR, Japanese Foundation for Cancer Research; KU, Keio University; PDAC, pancreatic ductal adenocarcinoma; UT, The University of Tokyo

We collected data on consecutive patients who underwent surgical resection of pancreatic malignancy between 2005 and 2017 at The Cancer Institute Hospital of Japanese Foundation for Cancer Research (JFCR), The University of Tokyo Hospital, and Keio University Hospital (all in Tokyo, Japan) from the GTK (Good to Know) Pancreatic Cancer consortium (Fig. 1).^29,30^ We included patients who had PC with available data on the ABO blood group. We excluded (1) patients with ductal adenocarcinoma variants, including intraductal papillary mucinous neoplasm-derived carcinoma, undifferentiated carcinoma, and colloid carcinoma, (2) patients with mixed tumors (e.g., mixed ductal-neuroendocrine carcinoma), (3) patients who had advanced cancer of other origins concomitantly at the time of the index surgery, and (4) patients with a 30-day or in-hospital mortality. The patients were followed up until death or the end of the follow-up period (31 May 2024), whichever came first.

Adjuvant chemotherapy regimens were categorized as follows: S-1-based chemotherapy (monotherapy or combination therapy) versus gemcitabine-based chemotherapy (monotherapy or combination therapy) versus others versus none. Gemcitabine-based adjuvant chemotherapy was administered as the first-line option during the early study period, and use of S-1-based adjuvant chemotherapy began after the JASPAC 01 trial, which demonstrated the superiority of S-1 over gemcitabine in this setting.^3^

The patients were categorized into a specific regimen group when at least one cycle (e.g., 6 weeks for S-1 and 4 weeks for gemcitabine) had been administered. Given our preliminary results showing a stronger survival association of the ABO blood group among patients receiving S-1-based chemotherapy, we categorized patients who received gemcitabine and S-1 into the S-1-based chemotherapy group and confirmed that the exclusion of this subgroup did not alter our findings substantially (data not shown). After postoperative recurrence, the patients who received gemcitabine-based adjuvant chemotherapy were generally treated with S-1-based chemotherapy, and vice versa.

Using a standardized study database constructed via Access software (Microsoft, Redmond, WA, USA), the participating physicians reviewed the medical charts and collected clinical data. Pathologists (M. Tak., Y.M., and M. Tan.), blinded to the clinical data, reviewed hematoxylin and eosin (H&E)-stained tissue sections of formalin-fixed paraffin-embedded tumor blocks and recorded the pathologic characteristics of pancreatic carcinomas. According to the guidelines of the Japan Pancreas Society,^31^ we classified the stroma type (medullary [scant stroma] or intermediate or scirrhous [abundant stroma]) and the resection margin status (R0 [no residual tumor cells on the dissection or cut surface], R1 [microscopic residual tumor], and R2 [macroscopic residual tumor]). The cancer stage was defined according to the eighth edition of the tumor-node-metastasis (TNM) staging system proposed by the Union for International Cancer Control (UICC).^32^ The resectability status was defined according to the anatomic criteria proposed by the National Comprehensive Cancer Network (NCCN).^33^ For adenocarcinoma cases, tumor differentiation was graded as well, moderate, or poor according to the World Health Organization classification.^34^

This study was designed and conducted in accordance with the Declaration of Helsinki guidelines. Given the retrospective design of the study, informed consent was obtained from all the patients on an opt-out basis. The study was approved by the ethics committees of all the participating centers and registered with the University Medical Information Network (UMIN) registry (registration no. UMIN000044027).

### Assessment of Blood Group Antigen Expression in Blood and Pancreatic Cancer Tissue

For the primary analysis, we collected serologically confirmed (not self-reported) data regarding the ABO blood group (O vs. A vs. B vs. AB), which was confirmed by duplicate tests at all centers. In cases with available tissue material at the JFCR (*n* = 654), we performed immunohistochemical analyses of blood group antigens A and B in the primary pancreatic carcinomas using tissue microarrays (the methods are detailed in Supplementary Methods).^30^ The primary monoclonal antibodies used were clone HE-193 (mouse: dilution, 1:200; Thermo Fisher Scientific, MA, USA; catalog no., MA1-19693) for antigen A and clone HEB-29 (mouse: dilution, 1:200; Thermo Fisher Scientific; catalog no. MA1-19691) for antigen B.^23^ A deep learning-based semi-automatic evaluation for staining positivity was performed.

### Assessment of Tumor Molecular Markers

We performed a droplet digital polymerase chain reaction for mutations in *KRAS* codons 12, 13, and 61, as previously described.^29^ The variant allele frequency of *KRAS* was calculated as the ratio of the number of *KRAS*-mutant droplets to the number of droplets containing the *KRAS*-mutant signal and/or the *KRAS*-wild-type signal. Tumors were classified as *KRAS*-mutant when the variant allele frequency was 1% or greater and otherwise as the *KRAS*-wild-type. We performed immunohistochemical analysis of the tumor expression status of CDKN2A (p16), TP53, and SMAD4, as previously described.^30^

### Statistical Analysis

We examined the association of the ABO blood group with survival time among the patients with PC, both overall and by specific adjuvant chemotherapy regimens. Disease-free survival (DFS) was defined as the time from index surgery to the first recurrence or death, whichever occurred first. When none of these end points were observed, the patients were censored at the time of the last cross-sectional imaging study. PC-specific survival was defined as the time from the index surgery to death from progressive PC, and the patients who died of causes other than PC or were alive at the last follow-up visit were censored.

In our primary analysis, the Cox proportional hazards regression model stratified by institutional cohort was used to calculate hazard ratios (HRs) and 95% confidence intervals (CIs) for disease-free and PC-specific survival according to each ABO blood group. To adjust for potential confounding factors, the multivariable Cox regression model initially included covariates described in the corresponding tables. Backward elimination with a threshold *P* value of 0.05 was performed to select variables for the final model. Patients with missing data (*KRAS* mutations, 0.5%; CDKN2A expression, 0.4%; TP53 expression, 0.5%; and SMAD4 expression, 0.6%) were included in the majority category of a given categorical covariate to limit the degrees of freedom of the models. We confirmed that the exclusion of cases with missing data for any of the variables did not substantially alter the results (data not shown).

The assumption of proportional hazards was generally satisfied by conducting the Wald test on a time-dependent covariate, which was the cross-product of the ABO blood group (A, B, or AB vs. O) and disease-free or PC-specific survival (*P* > 0.17). Statistical interactions were assessed using the likelihood ratio test on the cross-product of the ABO blood group (O vs. A vs. B vs. AB) and adjuvant chemotherapy regimens (S-1-based vs. gemcitabine-based vs. none) in the Cox regression model.

We calculated stratum-specific HRs based on a single regression model with reparameterization of the interaction term.^35^ We observed no statistically significant heterogeneity in the survival associations of the ABO blood group between the institutional cohorts using Cochran’s *Q* statistic^36^ (*P* > 0.07). Therefore, we pooled the cohorts for all analyses. Cumulative survival probabilities were estimated using the Kaplan–Meier product-limit method and compared using the log-rank test.

All statistical analyses were performed using SAS software (version 9.4; SAS Institute, Cary, NC, USA). We used the two-sided α level of 0.05 for statistical significance. Given the hypothesis-generating nature of this study, multiple comparisons were not considered. The results of the secondary analyses should be interpreted with caution.

## Results

Our multicenter cohort included 1153 patients with resected PC (Table 1; Fig. 1). The patients with blood group AB were more likely to have advanced cancer status and less likely to receive post-recurrence chemotherapy than the patients with the other blood groups. There were no statistically significant differences in the alterations of the four major driver genes (*KRAS* mutation and CDKN2A [p16], TP53, and SMAD4 expression) between the blood groups. During the median follow-up time of 85.0 months (25th to 75th percentiles, 56.0–113.8 months) for all the censored patients, 859 (75%) deaths occurred, including 787 (68%) PC-specific deaths.

**Table 1:** Clinical, pathologic, and molecular characteristics of patients with pancreatic cancer, overall and by ABO blood group

Characteristics^a^	All patients	ABO blood group				*P* Value
		O	A	B	AB	
	(*n* = 1153) *n* (%)	(*n* = 301) *n* (%)	(*n* = 475) *n* (%)	(*n* = 260) *n* (%)	(*n* = 117) *n* (%)	
Age (years)	67.1 ± 9.8	68.1 ± 9.4	66.4 ± 9.9	67.3 ± 9.6	67.3 ± 10.5	0.14
Sex						0.43
Female	479 (42)	128 (43)	193 (41)	102 (39)	56 (48)	
Male	674 (58)	173 (57)	282 (59)	158 (61)	61 (52)	
Year of diagnosis						0.88
2005–2010	333 (29)	85 (28)	142 (30)	68 (26)	38 (32)	
2011–2014	390 (34)	101 (34)	157 (33)	95 (37)	37 (32)	
2015–2017	430 (37)	115 (38)	176 (37)	97 (37)	42 (36)	
ASA physical status						0.29
I	216 (19)	50 (17)	102 (21)	46 (18)	18 (15)	
II	841 (73)	224 (74)	335 (71)	197 (76)	85 (73)	
III–IV	96 (8.3)	27 (9.0)	38 (8.0)	17 (6.5)	14 (12)	
CA19-9 (U/mL)	100	140	85	90	112	0.044
	(24–458)	(38–590)	(22–432)	(23–432)	(22–528)	
Tumor location						0.41
Head of the pancreas	734 (64)	181 (60)	305 (64)	174 (67)	74 (63)	
Body to tail of the pancreas	419 (36)	120 (40)	170 (36)	86 (33)	43 (37)	
Histologic type						0.67
Adenocarcinoma	1,123 (97)	293 (97)	464 (98)	254 (98)	112 (96)	
Adenosquamous carcinoma	30 (2.6)	8 (2.7)	11 (2.3)	6 (2.3)	5 (4.3)	
Tumor differentiation^b^						0.31
Well to moderate	656 (58)	173 (59)	271 (58)	139 (55)	73 (65)	
Poor	467 (42)	120 (41)	193 (42)	115 (45)	39 (35)	
Stroma type						0.84
Non-scirrhous	760 (66)	196 (65)	314 (66)	176 (68)	74 (63)	
Scirrhous	393 (34)	105 (35)	161 (34)	84 (32)	43 (37)	
Tumor size (cm)	3.4 ± 1.6	3.4 ± 1.4	3.3 ± 1.6	3.5 ± 1.7	3.6 ± 1.4	0.35
Positive lymph nodes						0.063
0	362 (31)	77 (26)	162 (34)	89 (34)	34 (29)	
1–3	461 (40)	128 (42)	193 (41)	99 (38)	41 (35)	
≥ 4	330 (29)	96 (32)	120 (25)	72 (28)	42 (36)	
UICC cancer stage						0.045
I	305 (26)	63 (21)	140 (29)	75 (29)	27 (23)	
II	500 (43)	142 (47)	205 (43)	108 (42)	45 (38)	
III	300 (26)	88 (29)	109 (23)	67 (26)	36 (31)	
IV	48 (4.2)	8 (2.7)	21 (4.4)	10 (3.9)	9 (7.7)	
NCCN resectability status						0.58
Resectable	868 (75)	218 (72)	366 (77)	191 (73)	93 (79)	
Borderline resectable	258 (22)	74 (25)	101 (21)	62 (24)	21 (18)	
Unresectable	27 (2.3)	9 (3.0)	8 (1.7)	7 (2.7)	3 (2.6)	
Resection margin status						0.085
R0	894 (78)	248 (82)	359 (76)	200 (77)	87 (74)	
R1	254 (22)	52 (17)	115 (24)	59 (23)	28 (24)	
R2	5 (0.4)	1 (0.3)	1 (0.2)	1 (0.4)	2 (1.7)	
Neoadjuvant chemotherapy^c^						0.048
None	1,003 (87)	247 (82)	429 (90)	221 (85)	106 (91)	
S-1-based	47 (4.1)	19 (6.3)	11 (2.3)	14 (5.4)	3 (2.6)	
Monotherapy	4 (0.4)	2 (0.7)	0	2 (0.8)	0	
Combination therapy	43 (3.7)	17 (5.7)	11 (2.3)	12 (4.6)	3 (2.6)	
Gemcitabine-based	76 (6.6)	27 (9.0)	23 (4.8)	20 (7.7)	6 (5.1)	
Monotherapy	7 (0.6)	5 (1.7)	2 (0.4)	0	0	
Combination therapy	69 (6.0)	22 (7.3)	21 (4.4)	20 (7.7)	6 (5.1)	
Others	27 (2.3)	8 (2.7)	12 (2.5)	5 (1.9)	2 (1.7)	
Adjuvant chemotherapy^c^						0.11
None	242 (21)	68 (23)	101 (21)	54 (21)	19 (16)	
S-1-based	471 (41)	122 (40)	195 (41)	112 (43)	42 (36)	
Monotherapy	439 (38)	113 (38)	186 (39)	105 (40)	35 (30)	
Combination therapy	32 (2.8)	9 (3.0)	9 (1.9)	7 (2.7)	7 (6.0)	
Gemcitabine-based	339 (29)	81 (27)	143 (30)	66 (25)	49 (42)	
Monotherapy	318 (28)	79 (26)	132 (28)	64 (25)	43 (37)	
Combination therapy	21 (1.8)	2 (0.7)	11 (2.3)	2 (0.8)	6 (5.1)	
Others	101(8.8)	30 (10)	36 (7.6)	28 (11)	7 (6.0)	
Recurrence pattern^d^						0.23
Local	179 (22)	43 (20)	87 (26)	35 (20)	14 (19)	
Metastatic	618 (78)	174 (80)	247 (74)	138 (80)	59 (81)	
Chemotherapy after recurrence^c,d^						0.006
None	179 (22)	42 (19)	73 (22)	39 (23)	25 (34)	
S-1-based	201 (25)	53 (24)	83 (25)	42 (24)	23 (32)	
Monotherapy	180 (23)	47 (22)	76 (23)	34 (20)	23 (32)	
Combination therapy	21 (2.6)	6 (2.8)	7 (2.1)	8 (4.6)	0	
Gemcitabine-based	365 (46)	115 (53)	146 (44)	83 (48)	21 (29)	
Monotherapy	172 (22)	59 (27)	66 (20)	36 (21)	11 (15)	
Combination therapy	193 (24)	56 (26)	80 (24)	47 (27)	10 (14)	
Others	52 (6.5)	7 (3.2)	32 (9.6)	9 (5.2)	4 (5.5)	
*KRAS* mutation						0.23
Wild type	23 (2.0)	9 (3.0)	5 (1.1)	7 (2.7)	2 (1.7)	
Mutant	1,124 (98)	292 (97)	465 (99)	252 (97)	115 (98)	
CDKN2A (p16) expression						0.73
Intact	168 (15)	45 (15)	71 (15)	39 (15)	13 (11)	
Lost	980 (85)	255 (85)	400 (85)	221 (85)	104 (89)	
TP53 expression						0.090
Intact	584 (51)	170 (57)	223 (47)	134 (52)	57 (49)	
Aberrant	563 (49)	130 (43)	247 (53)	126 (48)	60 (51)	
SMAD4 expression						0.96
Intact	513 (45)	132 (44)	208 (44)	119 (46)	54 (46)	
Lost	633 (55)	167 (56)	262 (56)	141 (54)	63 (54)	

ASA, American Society of Anesthesiologists; CA19-9, carbohydrate antigen 19-9; NCCN, National Comprehensive Cancer Network; UICC, Union for International Cancer Control

^a^Data are presented as mean ± standard deviation, median (25th to 75th percentile), or number of patients (%). Percentage indicates the proportion of patients with a specific clinical, pathologic, or molecular characteristic in all cases or in each stratum of the ABO blood group. The total percentage may not be equal to 100% due to rounding. To compare the characteristics between the groups, the chi-square test or Fisher’s exact test (as appropriate) was used for categorical variables, and the analysis of variance or the Kruskal-Wallis test (as appropriate) was used for continuous variables

^b^Tumor differentiation was assessed only for adenocarcinomas

^c^Four groups (S-1-based chemotherapy vs. gemcitabine-based chemotherapy vs. others vs. none) were compared

^d^Patients who underwent recurrence were considered. Data on chemotherapy regimens after recurrence were missing in 17 cases

**Fig. 2:**
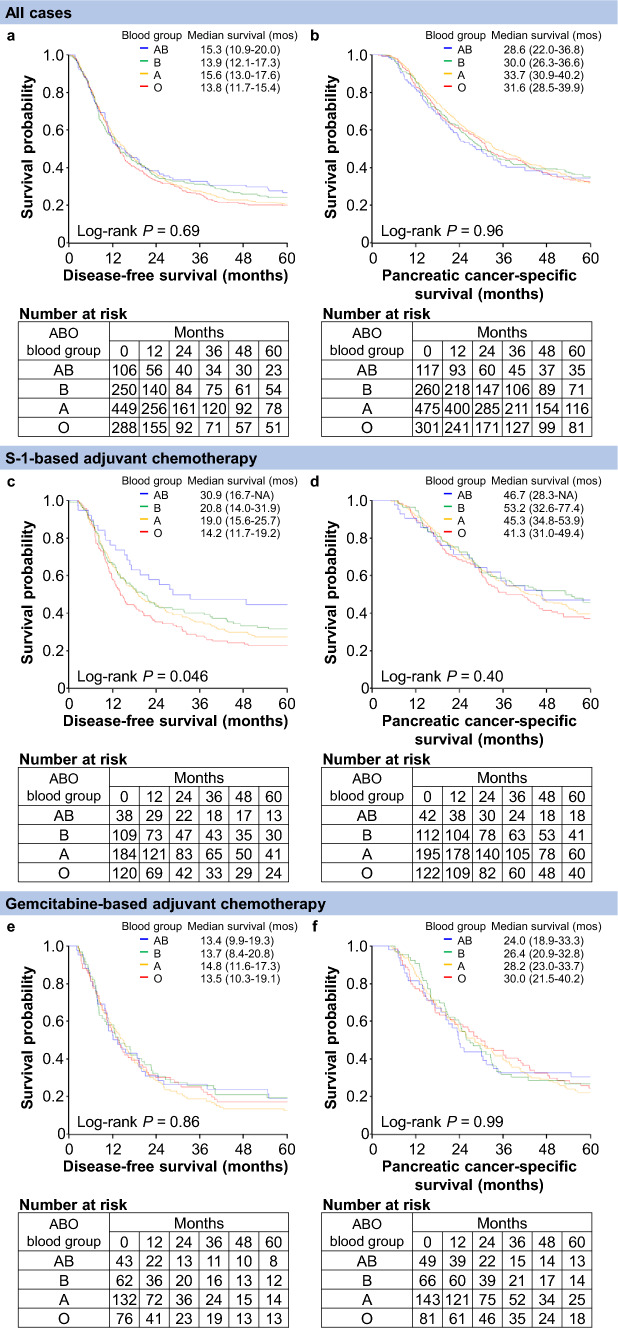

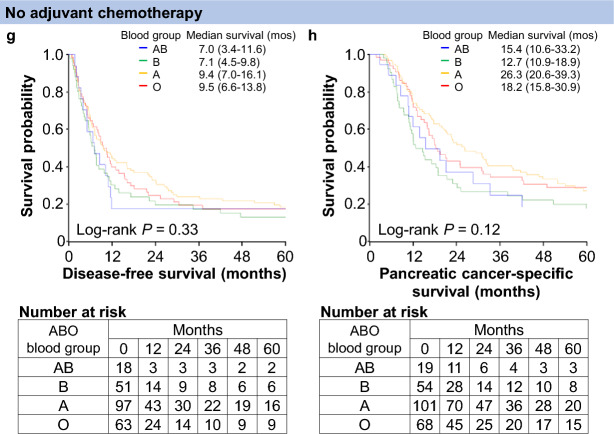
Kaplan–Meier curves for disease-free and pancreatic cancer-specific survival times among patients with pancreatic cancer according to the ABO blood group, overall and by adjuvant chemotherapy regimens. **a** and **b**. Disease-free and pancreatic cancer-specific survival times, respectively, among all patients. **c** and **d** Disease-free and pancreatic cancer-specific survival times, respectively, among patients who received S-1-based adjuvant chemotherapy. **e** and **f** Disease-free and pancreatic cancer-specific survival times, respectively, among patients who received gemcitabine-based adjuvant chemotherapy. **g** and **h** Disease-free and pancreatic cancer-specific survival times, respectively, among patients who did not receive adjuvant chemotherapy. Survival times are presented as medians (95% confidence intervals). NA, not available

In our primary analyses, the ABO blood group was not associated with disease-free (*P* = 0.91) or PC-specific (*P* = 0.93) survival (Table 2). For DFS, compared with patients with blood group O, the patients with blood groups A, B, and AB had multivariable HRs of 0.99 (95% CI, 0.84–1.18), 1.04 (95% CI, 0.86–1.26), and 0.95 (95% CI, 0.73–1.23), respectively, and the corresponding multivariable HRs for PC-specific survival were 0.97 (95% CI, 0.81–1.15), 1.03 (95% CI, 0.83–1.26), and 0.99 (95% CI, 0.76–1.30), respectively. In the analyses of the dichotomized blood groups (O vs. non-O), we consistently observed null survival associations of the blood group (multivariable HRs, 1.00 [95% CI, 0.86–1.17] for DFS and 0.99 [95% CI, 0.84–1.16] for PC-specific survival). Kaplan–Meier survival curves demonstrated similar associations of the ABO blood group with disease-free and PC-specific survival (Fig. 2a and b).

**Table 2:** ABO blood group and survival among patients with pancreatic cancer, overall and by adjuvant chemotherapy regimens

	Disease-free survival				Pancreatic cancer-specific survival			
	No. of patients	No. of events	Univariable HR (95% CI)	Multivariable HR^a^ (95% CI)	No. of patients	No. of events	Univariable HR (95% CI)	Multivariable HR^a^ (95% CI)
All cases								
ABO blood group								
O	288	235	1 (referent)	1 (referent)	301	209	1 (referent)	1 (referent)
A	449	368	0.95 (0.80–1.12)	0.99 (0.84–1.18)	475	325	0.93 (0.78–1.11)	0.97 (0.81–1.15)
B	250	199	0.91 (0.76–1.11)	1.04 (0.86–1.26)	260	174	0.94 (0.76–1.14)	1.03 (0.83–1.26)
AB	106	81	0.85 (0.66–1.10)	0.95 (0.73–1.23)	117	79	0.96 (0.74–1.24)	0.99 (0.76–1.30)
* P* Value			0.61	0.91			0.87	0.93
S-1-based								
ABO blood group								
O	120	96	1 (referent)	1 (referent)	122	83	1 (referent)	1 (referent)
A	184	138	0.83 (0.64–1.08)	0.94 (0.72–1.22)	195	121	0.87 (0.66–1.15)	0.99 (0.75–1.33)
B	109	77	0.76 (0.57–1.03)	0.96 (0.71–1.30)	112	63	0.76 (0.55–1.06)	0.94 (0.67–1.31)
AB	38	22	0.54 (0.34–0.86)	0.63 (0.39–1.00)	42	23	0.74 (0.47–1.18)	0.83 (0.52–1.32)
Gemcitabine-based								
ABO blood group								
O	76	67	1 (referent)	1 (referent)	81	64	1 (referent)	1 (referent)
A	132	118	1.07 (0.79–1.45)	1.11 (0.82–1.50)	143	111	1.03 (0.76–1.40)	1.01 (0.74–1.38)
B	62	55	0.95 (0.66–1.36)	0.90 (0.63–1.29)	66	53	0.97 (0.67–1.40)	0.83 (0.57–1.20)
AB	43	36	0.94 (0.63–1.42)	0.95 (0.63–1.42)	49	38	1.01 (0.67–1.50)	0.90 (0.59–1.35)
None								
ABO blood group								
O	63	51	1 (referent)	1 (referent)	68	46	1 (referent)	1 (referent)
A	97	84	0.86 (0.61–1.23)	0.99 (0.69–1.41)	101	72	0.85 (0.59–1.23)	0.97 (0.67–1.42)
B	51	46	1.24 (0.83–1.85)	1.65 (1.09–2.48)	54	42	1.42 (0.94–2.17)	1.96 (1.27–3.01)
AB	18	16	1.35 (0.77–2.38)	1.79 (1.01–3.17)	19	14	1.21 (0.66–2.20)	1.78 (0.97–3.26)
* P*_interaction_ Value^b^			0.065	0.011			0.16	0.008

CI, confidence interval; HR, hazard ratio

^a^In addition to the ABO blood group, the multivariable Cox regression model initially included age at surgery (continuous), sex (female vs. male), year of diagnosis (continuous), the American Society of Anesthesiologists physical status (continuous), carbohydrate antigen 19-9 (≤37 U/mL [normal range] vs. 38–500 U/mL vs. >500 U/mL), tumor location (head vs. body/tail of the pancreas), histologic type with tumor differentiation (well/moderately differentiated vs. poorly differentiated vs. adenosquamous), stroma type (non-scirrhous vs. scirrhous), cancer stage (I vs. II vs. III/IV), resectability status (resectable vs. borderline resectable/unresectable), resection margin status (R0 vs. R1/2), receipt of neoadjuvant chemotherapy (yes vs. no), type of adjuvant chemotherapy (S-1-based vs. gemcitabine-based vs. others vs. none, not included for the stratified analyses), *KRAS* mutation (wild type vs. mutant), CDKN2A expression (intact vs. lost), TP53 expression (intact vs. aberrant), and SMAD4 expression (intact vs. lost). Backward elimination with a threshold *P* value of 0.05 was performed to select variables for the final models. In the analysis of the total study population, the remaining variables in the final model were carbohydrate antigen 19-9, histologic type with tumor differentiation, cancer stage, resection margin status, type of adjuvant chemotherapy, TP53 expression (only for pancreatic cancer-specific survival), and SMAD4 expression. In the stratified analyses, the remaining variables in the final model were carbohydrate antigen 19-9, histologic type with tumor differentiation, cancer stage, resection margin status, TP53 expression (only for pancreatic cancer-specific survival), and SMAD4 expression (only for disease-free survival)

^b^*P*_interaction_ value was calculated by performing a likelihood ratio test for the cross-product terms (ABO blood groups vs. adjuvant chemotherapy regimens)

The associations of ABO blood group with disease-free and PC-specific survival differed according to the adjuvant chemotherapy regimen (*P*_interaction_ = 0.011 and 0.008, respectively; Table 2). Among the patients without chemotherapy, blood groups A, B, and AB (vs. O) had multivariable HRs for DFS of 0.99 (95% CI, 0.69–1.41), 1.65 (95% CI, 1.09–2.48), and 1.79 (95% CI, 1.01–3.17), respectively. The corresponding HRs for PC-specific survival were 0.97 (95% CI, 0.67–1.42), 1.96 (95% CI, 1.27–3.01), and 1.78 (95% CI, 0.97–3.26), respectively.

Among the patients receiving S-1-based chemotherapy, an inverse relationship was observed, with multivariable HRs for DFS of 0.94 (95% CI, 0.72–1.22) for blood group A, 0.96 (95% CI, 0.71–1.30), for blood group B, and 0.63 (95% CI, 0.39–1.00) for blood group AB (vs. O). When we examined how each of the covariates influenced the statistical interaction by adding it to the univariable model, we found that cancer stage was the strongest effect for both disease-free and PC-specific survival. The Kaplan–Meier survival curves demonstrated consistent results (Fig. 2c–h). We found that S-1-based chemotherapy was associated with longer DFS than gemcitabine-based chemotherapy for the patients with blood groups A and AB, but not for the patients with blood groups O and B (Table 3).

**Table 3:** Adjuvant chemotherapy regimens (S-1-based vs. gemcitabine-based) and survival among patients with pancreatic cancer in the strata of the ABO blood group

	Disease-free survival				Pancreatic cancer-specific survival			
	No. of patients	No. of events	Univariable HR (95% CI)	Multivariable HR^a^ (95% CI)	No. of patients	No. of events	Univariable HR (95% CI)	Multivariable HR^a^ (95% CI)
Blood group O								
Adjuvant chemotherapy								
Gemcitabine-based	76	67	1 (referent)	1 (referent)	81	64	1 (referent)	1 (referent)
S-1-based	120	96	0.85 (0.62–1.17)	0.82 (0.59–1.12)	122	83	0.72 (0.52–0.99)	0.69 (0.49–0.95)
Blood group A								
Adjuvant chemotherapy								
Gemcitabine-based	132	118	1 (referent)	1 (referent)	143	111	1 (referent)	1 (referent)
S-1-based	184	138	0.65 (0.51–0.83)	0.70 (0.55–0.90)	195	121	0.60 (0.46–0.77)	0.68 (0.52–0.88)
Blood group B								
Adjuvant chemotherapy								
Gemcitabine-based	62	55	1 (referent)	1 (referent)	66	53	1 (referent)	1 (referent)
S-1-based	109	77	0.67 (0.48–0.95)	0.84 (0.59–1.20)	112	63	0.55 (0.38–0.79)	0.70 (0.48–1.01)
Blood group AB								
Adjuvant chemotherapy								
Gemcitabine-based	43	36	1 (referent)	1 (referent)	49	38	1 (referent)	1 (referent)
S-1-based	38	22	0.47 (0.28–0.80)	0.55 (0.32–0.93)	42	23	0.52 (0.31–0.87)	0.65 (0.39–1.10)

CI, confidence interval; HR, hazard ratio

^a^In addition to the adjuvant chemotherapy regimens, the multivariable Cox regression model initially included age at surgery (continuous), sex (female vs. male), year of diagnosis (continuous), the American Society of Anesthesiologists physical status (continuous), carbohydrate antigen 19-9 (≤37 U/mL [normal range] vs. 38–500 U/mL vs. >500 U/mL), tumor location (head vs. body/tail of the pancreas), histologic type with tumor differentiation (well/moderately differentiated vs. poorly differentiated vs. adenosquamous), stroma type (non-scirrhous vs. scirrhous), cancer stage (I vs. II vs. III/IV), resectability status (resectable vs. borderline resectable/unresectable), resection margin status (R0 vs. R1/2), receipt of neoadjuvant chemotherapy (yes vs. no), *KRAS* mutation (wild type vs. mutant), CDKN2A expression (intact vs. lost), TP53 expression (intact vs. aberrant), and SMAD4 expression (intact vs. lost). Backward elimination with a threshold *P* value of 0.05 was performed to select variables for the final models. The remaining variables in the final model were age at surgery (only for pancreatic cancer-specific survival), carbohydrate antigen 19-9, cancer stage, resection margin status, and receipt of neoadjuvant chemotherapy

**Fig. 3:**
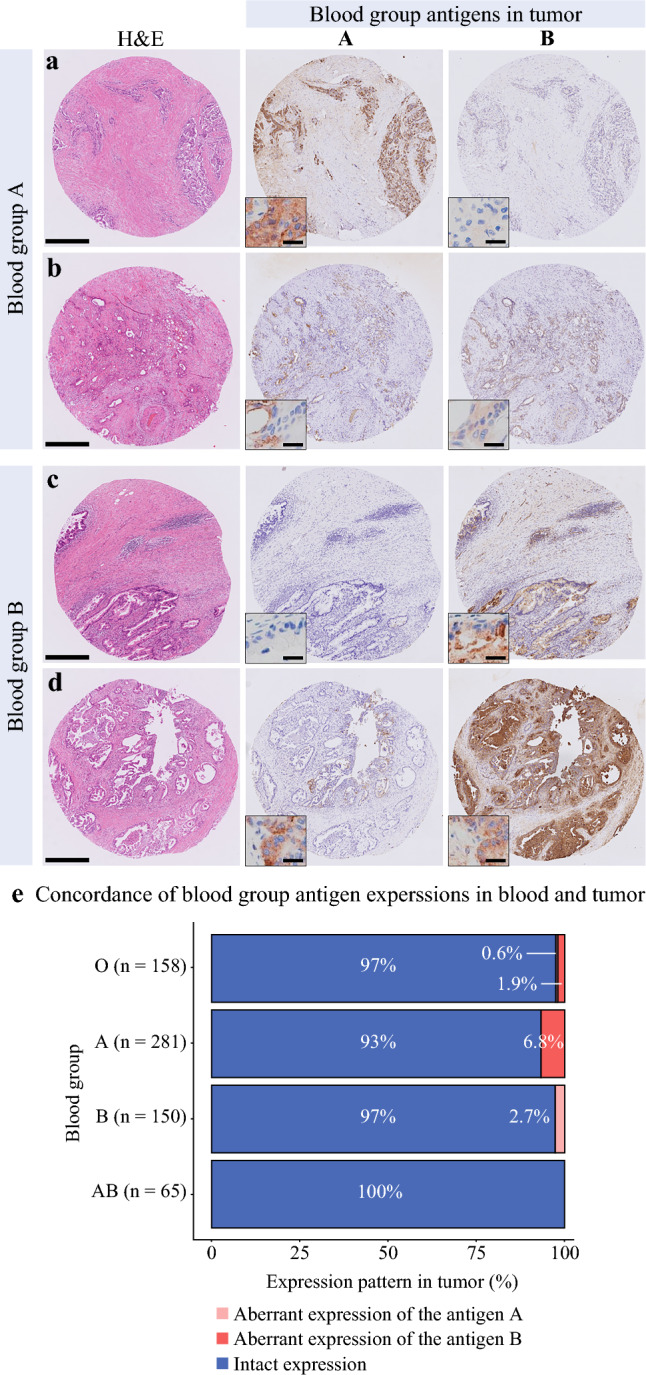
Immunohistochemistry analyses of the expression status of blood group antigens A and B in pancreatic cancer. **a** Case of blood group A and no expression of blood group antigen B in the tumor. **b** Case of blood group A and aberrant expression of blood group antigen B in the tumor. **c** Case of blood group B and no expression of blood group antigen A in the tumor. **d** Case of blood group B and aberrant expression of blood group antigen A in the tumor. **e** Concordance of blood group antigen expressions in blood and tumor. Scale bars: 500 µm (*main image*) and 20 µm (*inset*). H&E, hematoxylin and eosin

We performed immunohistochemical analyses of blood group antigens A and B in the PC tissue of a subset of patients (*n* = 654; Fig. 3). Aberrant positive expression of antigens A and B was observed in 5 (0.8%) and 22 (3.4%) patients, respectively (Fig. 3e). Compared with tumors that had intact antigen B expression, tumors with aberrant expression were more likely to be poorly differentiated and exhibit intact CDKN2A expression (Table 4). Similar results from the analysis of serologic blood groups in the total study population were observed in terms of survival outcomes (Table S1).

**Table 4:** Clinical, pathologic, and molecular characteristics of patients with pancreatic cancer by tumor expression status of the blood group antigens A and B (intact vs. aberrant)

Characteristics^a^	Antigen A (tumor)^b^		*P* Value	Antigen B (tumor)^b^		*P* Value
	Intact	Aberrant		Intact	Aberrant	
	(*n* = 303) *n* (%)	(n = 5) *n* (%)		(*n* = 417) *n* (%)	(*n* = 22) *n* (%)	
Age (years)	66.9 ± 9.1	65.4 ± 3.6	0.72	66.2 ± 9.3	64.2 ± 12.0	0.33
Sex			> 0.99			0.040
Female	126 (42)	2 (40)		188 (45)	5 (23)	
Male	177 (58)	3 (60)		229 (55)	17 (77)	
Year of diagnosis			0.28			0.001
2005–2010	93 (31)	0		138 (33)	1 (4.6)	
2011–2014	95 (31)	3 (60)		135 (32)	5 (23)	
2015–2017	115 (38)	2 (40)		144 (35)	16 (73)	
ASA physical status			> 0.99			> 0.99
I	61 (20)	1 (20)		89 (21)	5 (23)	
II	221 (73)	4 (80)		300 (72)	16 (73)	
III–IV	21 (6.9)	0		28 (6.7)	1 (4.6)	
CA19-9 (U/mL)	181	112	0.62	148	56	0.092
	(25–781)	(14–287)		(26–681)	(16–216)	
Tumor location			0.66			0.25
Head of the pancreas	191 (63)	4 (80)		277 (66)	12 (55)	
Body to tail of the pancreas	112 (37)	1 (20)		140 (34)	10 (45)	
Histologic type			> 0.99			> 0.99
Adenocarcinoma	294 (97)	5 (100)		405 (97)	22 (100)	
Adenosquamous carcinoma	9 (3.0)	0		12 (2.9)	0	
Tumor differentiation^c^			0.36			0.004
Well to moderate	186 (63)	2 (40)		270 (67)	8 (36)	
Poor	108 (37)	3 (60)		135 (33)	14 (64)	
Stroma type			0.62			0.49
Non-scirrhous	217 (72)	3 (60)		294 (70)	14 (64)	
Scirrhous	86 (28)	2 (40)		123 (30)	8 (36)	
Tumor size (cm)	3.6 ± 1.4	3.6 ± 1.0	0.96	3.6 ± 1.6	3.0 ± 1.4	0.085
Positive lymph nodes			0.33			0.70
0	81 (27)	3 (60)		125 (30)	5 (23)	
1–3	131 (43)	1 (20)		174 (42)	11 (50)	
≥ 4	91 (30)	1 (20)		118 (28)	6 (27)	
UICC cancer stage			0.70			0.79
I	59 (19)	2 (40)		100 (24)	4 (18)	
II	148 (49)	2 (40)		192 (46)	11 (50)	
III	80 (26)	1 (20)		104 (25)	5 (23)	
IV	16 (5.3)	0		21 (5.0)	2 (9.1)	
NCCN resectability status			> 0.99			0.48
Resectable	207 (68)	4 (80)		295 (71)	18 (82)	
Borderline resectable	85 (28)	1 (20)		113 (27)	4 (18)	
Unresectable	11 (3.6)	0		9 (2.2)	0	
Resection margin status			> 0.99			0.61
R0	246 (81)	4 (80)		328 (79)	19 (86)	
R1	56 (18)	1 (20)		88 (21)	3 (14)	
R2	1 (0.3)	0		1 (0.2)	0	
Neoadjuvant chemotherapy^d^			> 0.99			0.37
None	259 (85)	5 (100)		370 (89)	19 (86)	
S-1-based	5 (1.7)	0		2 (0.5)	0	
Monotherapy	2 (0.7)	0		0	0	
Combination therapy	3 (1.0)	0		2 (0.5)	0	
Gemcitabine-based	37 (12)	0		40 (9.6)	2 (9.1)	
Monotherapy	4 (1.3)	0		6 (1.4)	0	
Combination therapy	33 (11)	0		34 (8.2)	2 (9.1)	
Others	2 (0.7)	0		5 (1.2)	1 (4.6)	
Adjuvant chemotherapy^d^			0.17			0.23
None	63 (21)	0		89 (21)	3 (14)	
S-1-based	140 (46)	5 (100)		186 (45)	15 (68)	
Monotherapy	127 (42)	4 (80)		170 (41)	15 (68)	
Combination therapy	13 (4.3)	1 (20)		16 (3.8)	0	
Gemcitabine-based	96 (32)	0		137 (33)	4 (18)	
Monotherapy	94 (31)	0		130 (31)	3 (14)	
Combination therapy	2 (0.7)	0		7 (1.7)	1 (4.6)	
Others	4 (1.3)	0		5 (1.2)	0	
Recurrence pattern^e^			0.54			0.54
Local	38 (17)	1 (25)		65 (21)	2 (13)	
Metastatic	180 (83)	3 (75)		241 (79)	14 (88)	
Chemotherapy after recurrence^d,e^			0.51			0.43
None	41 (19)	0		58 (19)	4 (25)	
S-1-based	51 (23)	0		67 (22)	1 (6.3)	
Monotherapy	44 (20)	0		62 (20)	1 (6.3)	
Combination therapy	7 (3.2)	0		5 (1.6)	0	
Gemcitabine-based	114 (53)	4 (100)		152 (50)	10 (63)	
Monotherapy	56 (26)	2 (50)		81 (27)	2 (13)	
Combination therapy	58 (27)	2 (50)		71 (23)	8 (50)	
Others	12 (5.5)	0		29 (9.5)	1 (6.3)	
*KRAS* mutation			> 0.99			> 0.99
Wild type	7 (2.3)	0		4 (1.0)	0	
Mutant	296 (98)	5 (100)		413 (99)	22 (100)	
CDKN2A (p16) expression			0.59			0.040
Intact	63 (21)	0		71 (17)	8 (36)	
Lost	240 (79)	5 (100)		346 (83)	14 (64)	
TP53 expression			0.37			0.090
Intact	151 (50)	4 (80)		210 (50)	7 (32)	
Aberrant	152 (50)	1 (20)		207 (50)	15 (68)	
SMAD4 expression			> 0.99			0.91
Intact	128 (42)	2 (40)		175 (42)	9 (41)	
Lost	174 (58)	3 (60)		241 (58)	13 (59)	

ASA, the American Society of Anesthesiologists; CA19-9, carbohydrate antigen 19-9; NCCN, National Comprehensive Cancer Network; UICC, Union for International Cancer Control

^a^Data are presented as mean ± standard deviation, median (25th to 75th percentile), or number of patients (%). Percentage indicates the proportion of patients with a specific clinical, pathologic, or molecular characteristic in each stratum of tumor expression status of the blood group antigen. The total percentage may not be equal to 100%, due to rounding. To compare the characteristics between the groups, the chi-square test or Fisher’s exact test (as appropriate) was used for categorical variables and Student’s *t* test or the Wilcoxon rank-sum test (as appropriate) was used for continuous variables

^b^Characteristics by tumor expression status of blood group antigen A were examined among patients with blood group B or O. Characteristics by tumor expression status of blood group antigen B were examined among patients with blood group A or O

^c^Tumor differentiation was assessed only for adenocarcinomas

^d^Four groups (S-1-based chemotherapy vs. gemcitabine-based chemotherapy vs. others vs. none) were compared

^e^Patients who underwent recurrence were considered

## Discussion

In this large multi-institutional cohort of patients with resected PC, the survival association of the ABO blood group was not observed in the overall population but was observed in the patients receiving S-1-based adjuvant chemotherapy. The superiority of S-1-based chemotherapy over gemcitabine-based chemotherapy in an adjuvant setting was evident only in the patients with blood groups A and AB. The current study supports the potential of the ABO blood group as a biomarker that predicts postoperative survival outcomes for patients receiving adjuvant S-1 and identifies patient subgroups likely to benefit from S-1-based adjuvant chemotherapy. Our comprehensive set of clinical, pathologic, and molecular parameters enabled us to adjust for potential confounding factors rigorously in the multivariable models. This was important given that the adjustment for cancer stage (which was a clinically plausible confounding factor) resulted in a stronger interaction between the ABO blood group and adjuvant chemotherapy regimens in our survival analyses.

It is well established that individuals with the non-O blood groups have a higher risk for the development of PC than those with blood group O.^12–14^ However, the association between the ABO blood group and post-diagnostic survival outcomes of PC remains unclear. Despite the null survival associations of the ABO blood group reported in some studies,^19–21,37,38^ other studies have indicated that patients with the non-O blood groups have a worse prognosis after PC resection than patients with blood group O.^22,39^ Rahbari et al.^39^ reported that patients with blood group A had worse OS than patients with blood group O (multivariable HR comparing O to A: 0.78; 95% CI, 0.62–0.99). Tezuka et al.^22^ documented that the non-O blood group was an independent prognostic factor for OS with a multivariable HR of 1.58 (95% CI, 1.18–2.13).

In line with these findings, our survival analysis of patients without adjuvant chemotherapy showed that the presence of blood group antigens may result in shorter OS times, suggesting an aggressive phenotype of pancreatic neoplasms associated with blood group antigens. Interestingly, the current study highlighted the potentially different risks of tumor recurrence and mortality associated with the ABO blood group by adjuvant chemotherapy regimens. Among the patients who received S-1-based chemotherapy, the association of non-O blood groups showing high recurrence and mortality risks with no adjuvant chemotherapy was largely attenuated or reversed. In contrast, Tezuka et al.^22^ suggested shorter survival times associated with the non-O blood group for patients receiving adjuvant S-1. This discrepancy might have been due to different study populations, unmeasured confounding factors, and/or chance findings. Nonetheless, our multicenter study design with a large sample may ensure the generalizability of our findings. Our data support the potential of ABO blood group as a biomarker for responsiveness to S-1-based adjuvant chemotherapy and survival outcomes among a specific subgroup of patients with resected PC.

We validated the survival outcomes for patients with PC based on serologically confirmed blood groups through IHC analysis. Additionally, our molecular analyses suggest that aberrant overexpression of antigen B may result in distinctive molecular pathologic characteristics of PC (e.g*.*, poorly differentiated histology and intact CDKN2A expression). If validated, these findings will provide valuable evidence for new therapeutic targets for patients with PC.

In our study, the frequency of aberrant expression of either blood group antigen was 4.1%, which appears to be lower than the rates of 1% to 33% reported in the literature.^16,18,27,40^ We used a deep learning-based pipeline that enabled sensitive and objective annotation of IHC results, even in PC cases with a scant tumor area, potentially preventing failure in detecting positive cells. Therefore, our findings support the potential of a patient’s blood group as a reliable surrogate for the blood group antigen expression in PC cells. In contrast, loss of expression of blood group antigens in PC cells of patients with blood groups A, B, or AB was reported in 13% to 33% of cases.^16,18,27,40^ Our IHC-based analysis did not identify any tumors with complete loss of staining in the whole area. However, there appeared to be faint staining or focal loss of staining in some tumors. Therefore, further investigation is required to determine the role of the suppressed expression of blood group antigens in the prognosis of patients with PC.

The current study had several limitations. First, our multivariable-adjusted analyses may have had unmeasured confounding factors. Nonetheless, our multivariable models included a comprehensive set of clinical, pathologic, and molecular characteristics, including alterations in four major driver genes (*KRAS* mutation and CDKN2A, TP53, and SMAD4 expression), and the adjustments did not alter the results substantially. Second, there was a lack of gold-standard criteria for IHC assessment of blood group antigens A and B. Nonetheless, it is more likely that the misclassification of expression status would have driven our findings of a statistical interaction between blood group antigens and adjuvant chemotherapy toward the null hypothesis. Additionally, our survival data, based on the IHC-based assessment of blood group antigens, did not materially differ from those based on serologic blood group profiling. Finally, a vast majority of the study population was Japanese. Therefore, our findings should be validated in independent populations of different racial diversity. In addition, S-1 is used predominantly in Asian countries, and therefore, further research is warranted to examine the effect modification by the ABO blood group in adjuvant chemotherapy regimens other than S-1.

In summary, our large cohort study, integrated with molecular data on resected PC, suggests that the ABO blood group may be differentially associated with the risk of recurrence and mortality according to adjuvant chemotherapy regimens. Given the promising clinical data on the effectiveness of S-1 in the adjuvant setting of PC, future research is warranted to identify targetable molecular and immune parameters and to optimize treatment strategies for better clinical outcomes for patients with blood groups O and B, who are less likely to benefit from adjuvant S-1 therapy.

## Supplementary Information

Below is the link to the electronic supplementary material.

Supplementary file1 (DOCX 87 KB)
