# Supplementary material for: Blood Group Antigen Expression in Blood and Tumor in Relation to Survival Outcomes in Resected Pancreatic Cancer, Overall and by Adjuvant Chemotherapy Regimens
Source: Ann Surg Oncol. 2025 May 2;32(9):6477–91. doi: 10.1245/s10434-025-17289-7 (PMC12317920; doi:10.1245/s10434-025-17289-7)
Supplement: Supplementary file 1 — Supplementary file1 (DOCX 87 KB) [file 10434_2025_17289_MOESM1_ESM.docx]

**Supplementary Methods**

***Assessment of Blood Group Antigen Expression in Pancreatic Cancer Tissue***

In cases with available tissue material at the Japanese Foundation for Cancer Research (n = 654), we conducted immunohistochemical analyses of blood group antigens A and B in pancreatic cancer (PC) cells. We constructed tissue microarrays (TMAs) from surgical specimens of primary pancreatic carcinomas, as previously described.[^1^](#_ENREF_1) We included up to four tumor cores from each patient (approximately 2 mm in diameter) in a single TMA block and used 4-μm-thick sections for immunohistochemistry. The primary monoclonal antibodies used were clone HE-193 (mouse; dilution, 1:200; Thermo Fisher Scientific, MA, USA; catalog number, MA1-19693) for antigen A and clone HEB-29 (mouse; dilution, 1:200; Thermo Fisher Scientific; catalog number, MA1-19691) for antigen B.[^2^](#_ENREF_2) Pathologist (M. Tak.), blinded to other data, conducted all the immunohistochemical assessments of the tumors. A deep learning-based pipeline using tumor cells with nuclear or cytoplasmic staining as input data was constructed to assess the positivity of blood group antigens in PC cells. All hematoxylin and eosin- and immunohistochemically stained slides were digitized using a digital slide scanner (NanoZoomer S360; Hamamatsu Photonics, Hamamatsu, Japan) to generate whole-slide images. Whole-slide images were used to generate nonoverlapping 224 × 224-pixel patch images. Following the detection of the color 3,3’-diaminobenzidine (DAB) in the patch images, the pathologist annotated a randomly selected sample of 17,214 immunohistochemistry patches into 10 classes, including cancer and non-cancer labels, and retrained the neural network model (MobileNet-V3, pre-trained based on the ImageNet database) with the labeled images. Subsequently, all images with at least one DAB-positive object (> 50 pixels in size) were classified as tumor or non-tumor based on the developed model. The inferred results were assembled per TMA core and subjected to subsequent assessment of the aberrant expression in cancer cells. A decision on matched vs. unmatched per patch was made by referring to a patient’s blood group: *e.g.*, when antigen A was positive and antigen B was completely negative in a patient with blood group A, the patch was regarded as matched with no aberrant expression. If a core included 50 or more blood group-unmatched patches, it was carefully checked by two pathologists (M. Tak. and Y.M.) to examine whether the core truly included cancer cells with aberrant expression. Blood group-matched and DAB-negative cores were reviewed and confirmed by a pathologist (M. Tak.). All immunohistochemical image assessments were performed using Python version 3.9.

**References**

1. Masugi Y, Takamatsu M, Tanaka M, et al. Post-operative mortality and recurrence patterns in pancreatic cancer according to KRAS mutation and CDKN2A, p53, and SMAD4 expression. The journal of pathology. Clinical research. Sep 2023;9(5):339-353.
2. Wang C, Zhou J, Wang L, et al. ABO blood groups and expression of blood group antigens of epithelial ovarian cancer in Chinese women. Cancer medicine. Mar 2023;12(6):7498-7507.

Supplementary Table 1. Tumor expression status of the blood group antigens A and B and survival among patients with pancreatic cancer, overall and by adjuvant chemotherapy regimens

|  | Disease-free survival | | | |  | Pancreatic cancer-specific survival | | | |
| --- | --- | --- | --- | --- | --- | --- | --- | --- | --- |
|  | No. of patients | No. of events | Univariable  HR (95% CI) | Multivariable HR^a^ (95% CI) |  | No. of patients | No. of events | Univariable  HR (95% CI) | Multivariable HR^a^ (95% CI) |
| Blood group antigens (tumor) |  |  |  |  |  |  |  |  |  |
| None | 144 | 126 | 1 (referent) | 1 (referent) |  | 154 | 120 | 1 (referent) | 1 (referent) |
| A | 243 | 206 | 0.83 (0.67-1.04) | 0.85 (0.67-1.06) |  | 263 | 198 | 0.87 (0.69-1.09) | 0.92 (0.73-1.16) |
| B | 139 | 113 | 0.81 (0.63-1.05) | 0.91 (0.70-1.18) |  | 149 | 105 | 0.81 (0.62-1.05) | 0.89 (0.68-1.17) |
| A and B | 78 | 63 | 0.84 (0.62-1.14) | 0.95 (0.70-1.29) |  | 88 | 68 | 0.94 (0.70-1.26) | 0.97 (0.71-1.33) |
|  |  |  |  |  |  |  |  |  |  |
| *P* |  |  | 0.32 | 0.53 |  |  |  | 0.40 | 0.84 |
|  |  |  |  |  |  |  |  |  |  |
| **S-1-based** |  |  |  |  |  |  |  |  |  |
| Blood group antigens (tumor) |  |  |  |  |  |  |  |  |  |
| None | 66 | 57 | 1 (referent) | 1 (referent) |  | 68 | 50 | 1 (referent) | 1 (referent) |
| A | 110 | 82 | 0.72 (0.52-1.02) | 0.82 (0.58-1.17) |  | 118 | 75 | 0.76 (0.53-1.08) | 0.81 (0.56-1.16) |
| B | 69 | 49 | 0.67 (0.46-0.98) | 0.78 (0.53-1.15) |  | 72 | 41 | 0.64 (0.43-0.97) | 0.70 (0.46-1.06) |
| A and B | 40 | 27 | 0.67 (0.42-1.05) | 0.76 (0.48-1.21) |  | 44 | 28 | 0.85 (0.53-1.34) | 0.85 (0.53-1.35) |
|  |  |  |  |  |  |  |  |  |  |
| **Gemcitabine-based** |  |  |  |  |  |  |  |  |  |
| Blood group antigens (tumor) |  |  |  |  |  |  |  |  |  |
| None | 47 | 41 | 1 (referent) | 1 (referent) |  | 50 | 41 | 1 (referent) | 1 (referent) |
| A | 78 | 75 | 1.18 (0.80-1.72) | 1.16 (0.79-1.70) |  | 87 | 77 | 1.19 (0.81-1.73) | 1.12 (0.77-1.65) |
| B | 42 | 38 | 0.99 (0.64-1.55) | 0.95 (0.61-1.48) |  | 46 | 38 | 0.96 (0.62-1.50) | 0.85 (0.54-1.33) |
| A and B | 28 | 26 | 1.22 (0.75-2.00) | 1.07 (0.65-1.76) |  | 33 | 30 | 1.24 (0.77-1.99) | 0.98 (0.61-1.59) |
|  |  |  |  |  |  |  |  |  |  |
| **None** |  |  |  |  |  |  |  |  |  |
| Blood group antigens (tumor) |  |  |  |  |  |  |  |  |  |
| None | 28 | 26 | 1 (referent) | 1 (referent) |  | 33 | 27 | 1 (referent) | 1 (referent) |
| A | 53 | 47 | 0.44 (0.27-0.71) | 0.52 (0.32-0.84) |  | 56 | 44 | 0.59 (0.37-0.96) | 0.81 (0.50-1.33) |
| B | 27 | 25 | 0.82 (0.47-1.41) | 1.06 (0.61-1.85) |  | 30 | 25 | 1.17 (0.68-2.01) | 1.63 (0.93-2.85) |
| A and B | 10 | 10 | 0.96 (0.46-1.99) | 1.58 (0.76-3.32) |  | 11 | 10 | 0.72 (0.35-1.48) | 1.36 (0.64-2.88) |
|  |  |  |  |  |  |  |  |  |  |
| *P*_interaction_^b^ |  |  | 0.013 | 0.012 |  |  |  | 0.056 | 0.062 |
|  |  |  |  |  |  |  |  |  |  |

^a^ In addition to the expression status of the blood group antigens, the multivariable Cox regression model initially included age at surgery (continuous), sex (female vs. male), year of diagnosis (continuous), the American Society of Anesthesiologists physical status (continuous), carbohydrate antigen 19-9 (≤ 37 U/mL [normal range] vs. 38-500 U/mL vs. > 500 U/mL), tumor location (head vs. body/tail of the pancreas), histological type with tumor differentiation (well/moderately differentiated vs. poorly differentiated vs. adenosquamous), stroma type (non-scirrhous vs. scirrhous), cancer stage (I vs. II vs. III/IV), resectability status (resectable vs. borderline resectable / unresectable), resection margin status (R0 vs. R1/2), receipt of neoadjuvant chemotherapy (yes vs. no), type of adjuvant chemotherapy (S-1-based vs. gemcitabine-based vs. others vs. none, not included for the stratified analyses), *KRAS* mutation (wild type vs. mutant), CDKN2A expression (intact vs. lost), TP53 expression (intact vs. aberrant), and SMAD4 expression (intact vs. lost). Backward elimination with a threshold *P* of 0.05 was conducted to select variables for the final models.

^b^ *P*_interaction_ was calculated by conducting a likelihood ratio test for the cross-product terms (blood group antigen expression in tumor vs. adjuvant chemotherapy types).

Abbreviations: CI, confidence interval; HR, hazard ratio.
